# Effects of intercropping different quinoa cultivars on peanut rhizosphere microorganisms and yield in saline-alkali soil

**DOI:** 10.3389/fmicb.2025.1729353

**Published:** 2025-12-17

**Authors:** Xiaoyan Liang, Rao Fu, Chuanjie Chen, Meng Li, Kuihua Yi, Haiyang Zhang, Yinyu Gu, Jiajia Li

**Affiliations:** 1 Shandong Institute of Sericulture, Shandong Academy of Agricultural Sciences, Yantai, China; 2 National Center of Technology Innovation for Comprehensive Utilization of Saline-Alkali Land, Dongying, China; 3 Shandong Engineering Research Center of Functional Crop Germplasm Innovation and Cultivation Utilization, Yantai, China

**Keywords:** intercrop, quinoa cultivars, peanut, rhizosphere microorganisms, yield, saline-alkali soil

## Abstract

Intercropping is an effective ecological utilization strategy in saline-alkali land, however, the response of peanut rhizosphere microorganisms in saline-alkali soil to different quinoa cultivars used in intercropping is unclear. In this study, a field experiment was conducted to intercrop peanut (IXP, ILP and IQP) with three quinoa cultivars Xinli 3 (IXQ), Longli 4 (ILQ) and Qinling 2 (IQQ), which differed significantly in plant traits. Illumina-based 16S rRNA gene sequencing was used to investigate the microbial diversity of peanut rhizosphere and to explore the relationship between with environment. The peanuts primarily accumulated sodium (Na) in their roots, especially during the vegetative stage (17.5 g/kg), whereas all plant parts substantially accumulated Na in the reproductive stage. Actinobacteriota and Proteobacteria were the dominant bacterial phyla of peanut rhizosphere, accounting for over 40% of the total bacteria in each group; *norank_f__Geminicoccaceae* and *norank_f__norank_o__Vicinamibacterales* were the dominant bacterial genera among all treatments, each exceeding 3.40%. The genus *Arthrobacter* exhibited the most significant differences in relative abundance among the three quinoa cultivars. The strongest association between peanut rhizosphere microbiota and yield was found when intercropping with IXQ. Stochastic processes dominate the assembly of bacterial communities under intercropping, with IXP exhibiting the highest normalized stochasticity ratio: 68.69% during the vegetative growth stage and 81.11% during the reproductive growth stage. Variance partitioning analysis further showed that peanut rhizosphere bacteria were most strongly correlated with yield (36.1%), followed by nutrient uptake (33.5%) and soil chemical properties (26.6%). Taken together, different quinoa cultivars used for intercropping substantially affected the correlation between peanut rhizosphere microorganisms and soil chemical properties, peanut growth, nutrient uptake, and pod yield, with cultivar IXQ showing the best effects for intercropping with peanuts in saline-alkali soil. These findings provide new insight into the pivotal roles of plant–microbe–yield interactions in abiotic stress mitigation.

## 1 Introduction

Salinity is a common form of abiotic stress to plants (Gao et al., 2007). Due to overexploitation of land resources, soil salinization is gradually emerging as a major global eco-environmental hazard (Zhang and Wang, 2021). This salinization issue is having severe impacts on agricultural resources in China, where saline-alkali conditions significantly influence soil quality, leading to reduced land productivity (Yang, 2008). Numerous studies have illustrated that saline-alkali soil is detrimental to the growth and yield of major crops (Qadir et al., 2014). In particular, a high concentration of NaCl has been shown to affect the seed germination rate, preventing the establishment of seedlings and having a detrimental effect on crop yield (Shkolnik et al., 2019). Salinity also affects the abundance, composition, and function of soil microbial communities, indirectly affect plant growth. For example, it significantly reduces the abundance of nitrite-oxidizing bacteria and inhibits nitrite oxidation rates, which adverse to biogeochemical nitrogen cycling (Wang et al., 2024). Saline-alkali stress alters the community structure and physical–chemical properties of the rhizosphere soil, resulting in decreased soil enzyme and bacterial activities (Qu et al., 2022). Overall, saline-alkali soil significantly reduces soil quality and crop yields, thereby posing a substantial threat to the sustainable use of land resources. Salt-induced land degradation results in substantial economic losses, primarily through reduced agricultural productivity (Qadir et al., 2014; Cheng et al., 2023a). Therefore, comprehensive research is needed to establish strategies for the rational and efficient management of saline-alkali soil to ultimately improve soil quality, enhance productivity, and achieve sustainable land utilization (Yang, 2008). Compared to the more costly option of reconstituting saline land, selecting the most suitable crops and cultivation practices for saline-alkali conditions, such as intercropping, may be a more economical, practical, and environmentally friendly approach (Shao et al., 2025).

Intercropping is a widely utilized agricultural practice, which serves as the primary farming method in certain regions. Intercropping involves the simultaneous cultivation of two or more crop species or genotypes on the same plot of land. This approach enhances overall productivity and contributes to the sustainable development of agriculture (Brooker et al., 2015; Mousavi and Eskandari, 2011). Compared to monoculture, intercropping not only results in better soil quality, but also enhances crop productivity (Zhou et al., 2011), helps to diversify crop varieties, and enhances the utilization rate of land resources. These benefits can be attributed to the complementary resource utilization capacities among different crop varieties as well as their enhanced resistance to pests, diseases, and weeds (Li C. et al., 2023). A substantial body of research has demonstrated that intercropping in saline-alkali soils can significantly reduce soil salinity, mitigate crop salt stress, and consequently enhance agricultural productivity (Su et al., 2024; Song et al., 2025). However, intercropping may also cause interspecific ecological competition, resulting in a reduced yield of certain crop varieties compared to monoculture (Franco et al., 2015; Wang et al., 2022). In addition, the species or cultivars used in intercropping has a large impact on the outcome (Namatsheve et al., 2020). Thus, selective breeding for intercropping cultivars is an active area of research (Dubey et al., 2024). In particular, identifying optimal crop pairings and suitable cultivars that confer synergistic productivity benefits in intercropping configurations is an important research priority.

Quinoa (*Chenopodium quinoa* Willd.) is a nutrient-dense crop in the *Amaranthaceae* family with considerable market potential that is attracting increased research attention (Filho et al., 2017). Quinoa is well-adapted to climatic extremities, including frost hardiness, halophytic competence, xerophytic traits, and oligotrophic soil viability, underpinning its potential for cultivation in marginal lands (Angeli et al., 2020). Peanut (*Arachis hypogaea* L.) is an important oilseed and cash crop that is cultivated worldwide (Ci et al., 2021), which shows moderate salt tolerance (Shao et al., 2025). Therefore, peanut has been cultivated in saline-alkali soils in northern China to alleviate the shortage of land resources (Ci et al., 2021). Quinoa and peanut are “cool-loving” and “warm-loving” crops, respectively, with different sowing and growing periods. Our previous studies showed that early sowing of quinoa reduces the salt content of the soil surface, contributing to the alleviation of salt stress in peanut seedlings (Liang et al., 2025). Therefore, a quinoa/peanut relay intercropping system has potential as a promising new cropping pattern on saline soils.

The plant rhizosphere is the soil layer surrounding the roots at a distance of 1–3 mm, representing a microenvironment that is intricately shaped by the plant (Qu et al., 2020). Microorganisms play a crucial role in the ecological, physical, and chemical processes of a saline-alkali soil ecosystem (Yu et al., 2019). Therefore, different microorganisms also have potential to reduce the degree of soil salinization. For instance, specific beneficial microorganisms such as halophilic bacteria contribute to the restoration of degrading saline-alkali soil to enhance its sustainable utilization (Ding Y. et al., 2025). In addition, manipulation of rhizosphere microbiota diversity is critical for sustainable crop production (Yang et al., 2016). Crop species and the cropping pattern selected significantly influence the diversity of bacterial communities in rhizosphere soil, with greater diversity found in intercropping systems relative to monoculture regimes; thus, intercropping can be an effective strategy to enhance soil fertility (Yang et al., 2016). Cucumber and wheat intercropping could facilitate the growth of potential beneficial microorganisms in the rhizosphere soil of cucumber (Jin et al., 2020). Furthermore, the improvement of rhizosphere soil microorganisms under intercropping promoted soybean growth (Wang et al., 2024). Similarly, intercropping of millet (*Echinochloa frumentacea*) and leguminous forages on saline-alkali soil improved the proportion of beneficial microorganisms while promoting the diversity and richness of rhizosphere soil microorganisms, demonstrating the beneficial utilization of saline-alkali land resources (Cheng et al., 2023b).

Despite this strong evidence for the benefits of intercropping and rizhosphere microorganisms, most of the previous studies in this field have focused on the impact of different types of crops on rhizosphere microorganisms, whereas there has been limited research on the effects of intercropping different cultivars of the same crop. Quinoa is characterized by a broad genetic diversity, comprising remarkable varieties with a wide range of traits that lead to variations in life-cycle duration, saponin content, and plant height (Dehghanian et al., 2024). Consequently, we selected three quinoa cultivars with significant differences in plant traits and intercropped them with peanuts to investigate the impacts of the different cultivars on peanut rhizosphere microorganisms and their relationships with soil chemical properties, peanut agronomic characteristics, nutrient uptake, and peanut yield.

## 2 Materials and methods

### 2.1 Experimental site and soil sampling

In 2022, the experimental field for this study was established at the Yellow River Delta Modern Agriculture Experimental and Demonstration Base of Shandong Academy of Agricultural Sciences (118.37 °E, 37.18 °N), Shandong Province, China. The three quinoa varieties used for intercropping were Xingli-3 (IXQ), Longli-4 (ILQ), and Qingli-2 (IQQ), and the corresponding intercropping treatments of peanuts is IXP, ILP, and IQP, respectively. The cultivars IXQ and ILQ were provided by Gansu Academy of Agricultural Sciences (Lanzhou, China), whereas IQQ was provided by Qinghai Academy of Agricultural and Forestry Sciences (Xining, China). More details of the cultivars are provided in Supplementary Tables S1, S2 and Supplementary Figure S1.

The physicochemical properties of the topsoil (0–20 cm layer) of the experimental field were as follows: pH of 7.96, electrical conductivity (EC) of 768 μs/cm, 1.03 g/kg total nitrogen (TN), 739 mg/kg total phosphate (TP), 19.2 g/kg total potassium (TK), 50.2 mg/kg available nitrogen (AN), 27.1 mg/kg available phosphorus (AP), 120 mg/kg available potassium (AK), and 13.91 g/kg organic carbon (OC). The rhizosphere soil attached to the plant roots was gently shaken off and sieved through a 2-mm mesh. Four plants from different rows were pooled together as a replica and three replicates were established for each treatment. The soil samples were divided into two parts: one was air-dried for analyzing soil chemical properties and the other was immediately frozen in liquid nitrogen and stored at −80 °C for molecular analysis.

### 2.2 Soil chemical properties

Soil pH and EC were measured using a glass electrode at a soil:water ratio of 1:5 after shaking the equilibration for approximately 30 min, as described previously (Dick et al., 2000). Soil samples were sieved with 1-mm mesh to measure nitrate-nitrogen (NO_3_^−^-N), ammonium nitrogen (NH_4_^+^-N), and phosphate ion (PO_4_^3−^-P). In brief, NO_3_^−^-N, NH_4_^+^-N, and PO_4_^3−^-P were extracted with 2 mol/L KCl and measured with a continuous flow analyzer (AutoAnalyzer-AA3, Seal Analytical, Norderstedt, Germany). Soil samples passed through 0.25-mm mesh were used to determine soil total organic C according to the Walkey–Black method (Walkley and Black, 1934). Soil ion (K^+^, Na^+^) concentrations were analyzed using a flame atomic absorption spectrophotometer Cole-Parmer FF-200D (Cole-Parmer, Cambridge, UK) as described by Sun et al. (2022).

### 2.3 Soil microbial community analysis

Genomic DNA was extracted from the rhizosphere soil samples with the E. Z. N. A.® soil DNA Kit (Omega Bio-Tek, Norcross, GA, USA) and the quality was checked by a NanoDrop 2000 ultraviolet–visible spectrophotometer. The bacterial universal V3-V4 region of the 16S rRNA gene was amplified using polymerase chain reaction (PCR) with the primers 338F (5′-ACTCCTACGGGAGGCAGCAG-3′) and 806R (5′-GGACTACHVGGGTATCTAAT-3′). The PCR products were quantified using a Quantus™ fluorometer (Promega Corporation, Madison, WI, USA) after purification. The purified amplicons were mixed and sequenced by Majorbio Bio-Pharm Technology Co. Ltd. (Shanghai, China) on the Illumina MiSeq PE300 platform (Illumina Inc., San Diego, CA, USA). More details are provided in Supplementary Table S3. All sequences have been deposited in the National Center for Biotechnology Information SRA database under accession number PRJNA1287218.

### 2.4 Data analysis

Significant differences in mean values among treatments were determined with analysis of variance and Duncan’s method (*p <* 0.05) with SPSS Statistics software (version 26.0; IBM Corporation, New York, NY, USA). Bacterial *α*-diversity, *β*-diversity, and functional analyses were performed using the free online Majorbio Cloud Platform^1^. Specifically, α-diversity analysis was conducted with Mothur software (v.1.30.2), Venn and bar diagrams were constructed with an R (ggvenn and ggplot2) (v.3.3.1), hierarchical clustering analysis was performed with Qiime (2020.2.0), principal coordinates analysis (PCoA) and the normalized stochasticity ratio (NST) were determined with nst packages (3.1.10) in R packages (v.3.3.1), linear discriminant analysis effect size (LEfSe) was performed with the Galaxy tool^2^, and the Mantel test was performed with Qiime (2020.2.0). Heatmaps were constructed with the R (v.3.3.1) pheatmap package. Variance partitioning analysis (VPA) was performed with the R (v.3.3.1) vegan package (v.2.4.3). In addition, redundancy analysis (RDA) was conducted using R (v.2.4.3) rda. Finally, the potential functions of bacterial communities were predicted using the Phylogenetic Investigation of Communities by Reconstruction of Unobserved States (PICRUSt) tool. Data are presented as mean ± standard error, and statistically significant differences among the means of different treatments were determined at *p* < 0.05.

## 3 Results

### 3.1 Agronomic characters and Na accumulation of intercropped peanut with different quinoa variety

**Figure 1:**
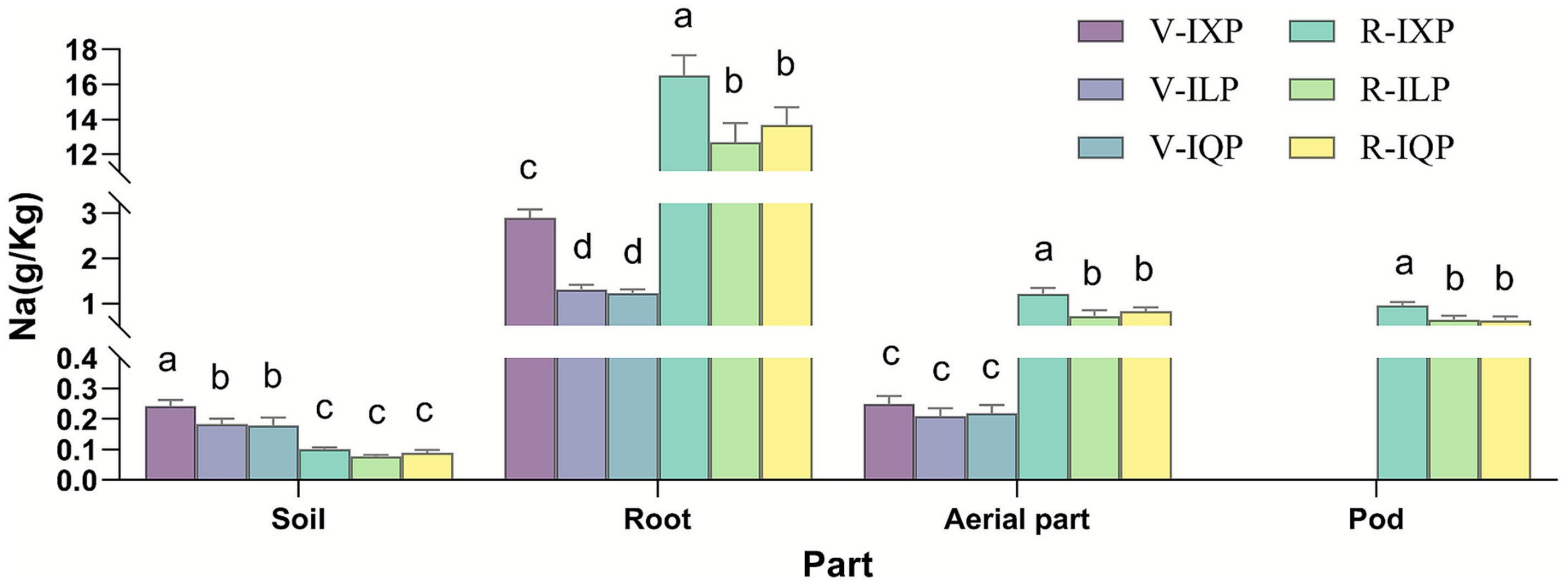
Na concentration of peanut and its rizhosphere soil when intercropped with different quinoa cultivars. V-IXP, the V stage of IXP (peanuts intercropped with quinoa cultivars IXQ); V-ILP, the V stage of ILP (peanuts intercropped with quinoa cultivars ILQ); V-IQP, the V stage of IQP (peanuts intercropped with quinoa cultivars IQQ); R-IXP, the R stage of IXP; R-ILP, the R stage of ILP; R-IQP, the R stage of IQP. Different lowercase letters indicate significant differences (*p* < 0.05).

The agronomic characters of intercropping quinoa and peanut were shown in Table 1. Regarding quinoa, the highest plant height and biomass were observed in IQQ, while the lowest were in IXQ. Regarding peanuts, IXP exhibited the highest plant height and biomass during the vegetative growth stage (V stage), however, during the reproductive growth stage (R stage), although IXP had the lowest height, it produced the highest biomass and pod yield. Regarding the V and R stages, the Na concentration in the peanut rhizosphere soil was lower during the R stage than during the V stage, however, the root and aboveground parts of peanuts are the opposite. Regarding the three cultivars, the Na concentration of IXP was significantly higher than ILP and IQP, which was consistent regardless of rhizosphere soil samples, plant part, and developmental stage (Figure 1). Regarding different plant parts, sodium concentrations in peanut roots were significantly higher than in the aboveground parts or pods (Supplementary Figure S2), additionally, only the aboveground parts of IQQ intercropped peanuts showed significantly higher sodium concentrations than pods (Supplementary Figure S3). Regarding sodium concentration changes, from V stage to R stage, there were no significant changes of Na concentration in soil and root among different cultivars, however, the Na concentration in both aerial part and whole plant of IXP were significantly higher compared to that of ILQ and IQQ (Supplementary Figure S4).

**Table 1:** Agronomic characters of quinoa and peanut in the vegetative (V) stage and reproductive (R) stage of peanut.

Plant	Treatment	V stage			R stage			
		Plant height (cm)	Dry weight of aboveground (g/plant)	Dry weight of underground (g/plant)	Plant height (cm)	Dry weight of aboveground (g/plant)	Dry weight of underground (g/plant)	Pod weight (g/plant)
Quinoa	IXQ	125c	152c	15.8c				
	ILQ	165b	188b	18.3b				
	IQQ	185a	222a	21.4a				
Peanut	IXP	9.67a	8.12a	0.723a	33.3b	12.6a	1.24a	47.2a
	ILP	9.42a	7.63a	0.681a	38.6a	11.2b	1.12b	40.5b
	IQP	8.42b	6.24b	0.594b	39.2a	10.0c	1.04c	32.7c

Data represent mean ± standard deviation (SD) of three biological replicates. Different lowercase letters within a row indicate significant differences (*P* < 0.05).

### 3.2 Alpha-diversity index analysis and bacterial composition

**Figure 2:**
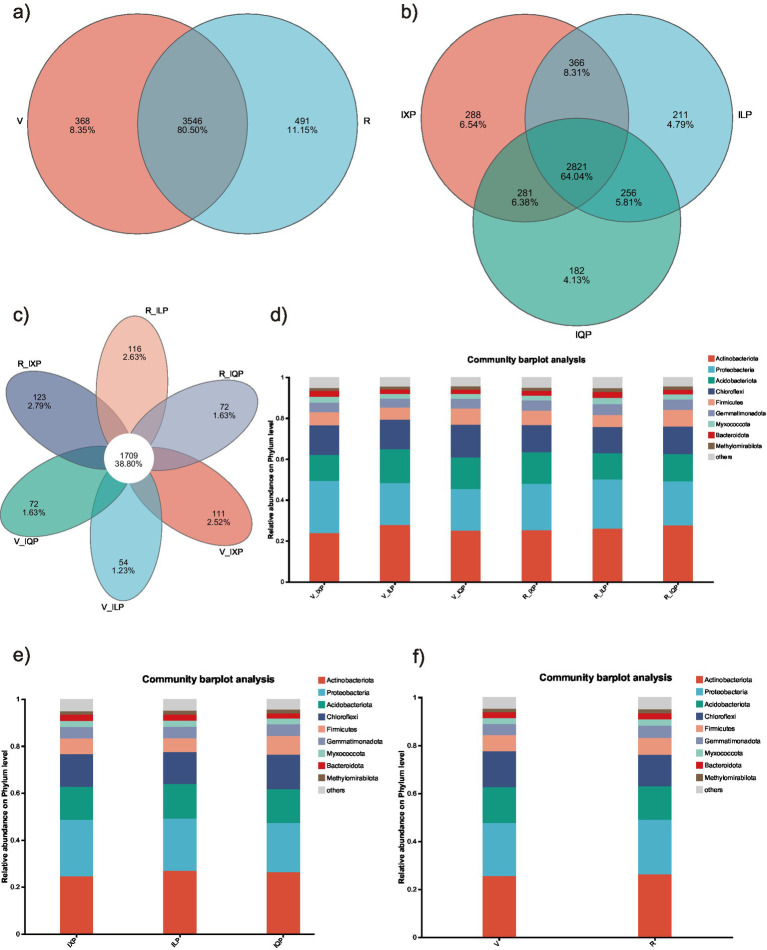
Composition of bacterial OTUs among different treatments. **(a)** Venn of two stages; **(b)** Venn of three quinoa cultivar conditions; **(c)** Venn of all treatments; **(d)** Bar of all treatments; **(e)** Bar of three quinoa cultivar conditions; **(f)** Bar of two stages.

Rarefaction curves (Supplementary Figure S5) combined with the estimated coverage values (Supplementary Table S4) suggested that the data were sufficient to reflect the bacterial diversity in the samples. There were no significant differences in the number of operational taxonomic units (OTUs) among groups (Supplementary Table S4); however, richness was significantly higher in the V_IXP and R_IXP treatments compared to that of the V_IQP treatment. The numbers of common and unique bacterial OTUs in the different treatments are shown in Figure 2. A total of 3,546 OTUs were common to the two developmental stages (V and R), and there was a greater number of unique OTUs (491) in the podding stage than in the seedling stage (368) (Figure 2a). Among the total 4,405 OTUs, 2,821 (64.04%) were shared by the three treatments, with a greater number of unique OTUs found for IXP than for ILP and IQP (Figure 2b). In addition, 1709 OTUs were shared by all treatments, with the highest number of unique OTUs found in the R_IXP treatment and the lowest number of OTUs found in the R_ILP treatment (Figure 2c).

The 4,405 OTUs were classified into 37 phyla, 123 classes, 282 orders, 453 families, 875 genera, and 1709 species. The bacterial communities identified in different samples at the phylum level are shown in Figure 2d. Actinobacteriota and Proteobacteria were the dominant bacterial phyla in all treatments, followed by Acdiobacteriota and Chloroflexi, and these four phyla occupied over 75% of the total, regardless of cultivars or stages (Figures 2d–f). *norank_f__Geminicoccaceae* was the dominant bacterial genus for the ILP and IXP cultivars, accounting for 5.02 and 5.21% of the bacterial population, respectively, and *norank_f__norank_o__Vicinamibacterales*, which was the dominant bacterial genus of IQP. In the meantime, *norank_f__Geminicoccaceae* and *norank_f__norank_o__Vicinamibacterales* were the dominant bacterial genera among all treatments and in the two developmental stages (Supplementary Figures S6–S8).

### 3.3 Beta-diversity analysis

**Figure 3:**
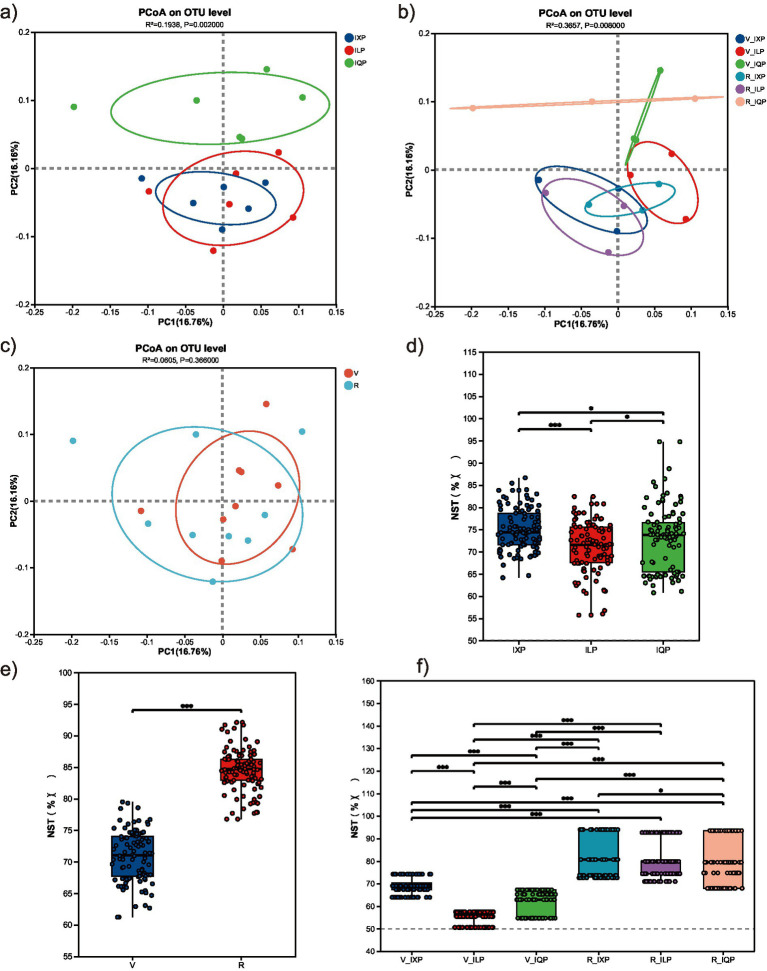
Microbial *β*-diversity and community structure. **(a)** PCoA of three quinoa cultivar conditions. **(b)** PCoA of all treatments. **(c)** PCoA of two stages. **(d)** NST of three quinoa cultivar conditions. **(e)** NST of two stages. **(f)** NST of all treatments.

PCoA based on the Bray–Curtis distance was conducted for the identified OTUs to reveal the main variations in bacterial community composition and abundance among the samples (Figures 3a–c). For all cultivars and treatments, PCoA identified two principal components explaining 16.76% (PC1) and 16.16% (PC2) of the variation in bacterial abundance among samples, respectively. The separation of the two developmental stages for the ILP and IQP was stronger than that for IXP (*R*^2^ = 0.194, *p* = 0.002). With respect to the cultivars, IXQ and ILQ conditions clustered together, while IQQ condition clustered separately, indicating that the microbial community structure was more similar between IXQ and ILQ conditions (*R*^2^ = 0.366, *p* = 0.008).

In addition, the NST was calculated to quantify the roles of deterministic and stochastic processes of bacterial communities under different cultivars and stages (Figures 3d–f). The NST values were above the 50% threshold for bacterial communities in the IXP, ILP, and IQP, with an average of 74.98, 71.02, and 72.87%, respectively, indicating that stochastic processes dominated the assembly of bacterial communities. Significant differences were observed among the three cultivars, with the highest species stochastic dispersal found for the IXP group. A similar result was observed for the different developmental stages. In both stages, the NST values of the bacterial communities were over 50%, with significantly higher values found for the R stage than for the V stage (*p* < 0.001).

### 3.4 Differential bacteria analysis

**Figure 4:**
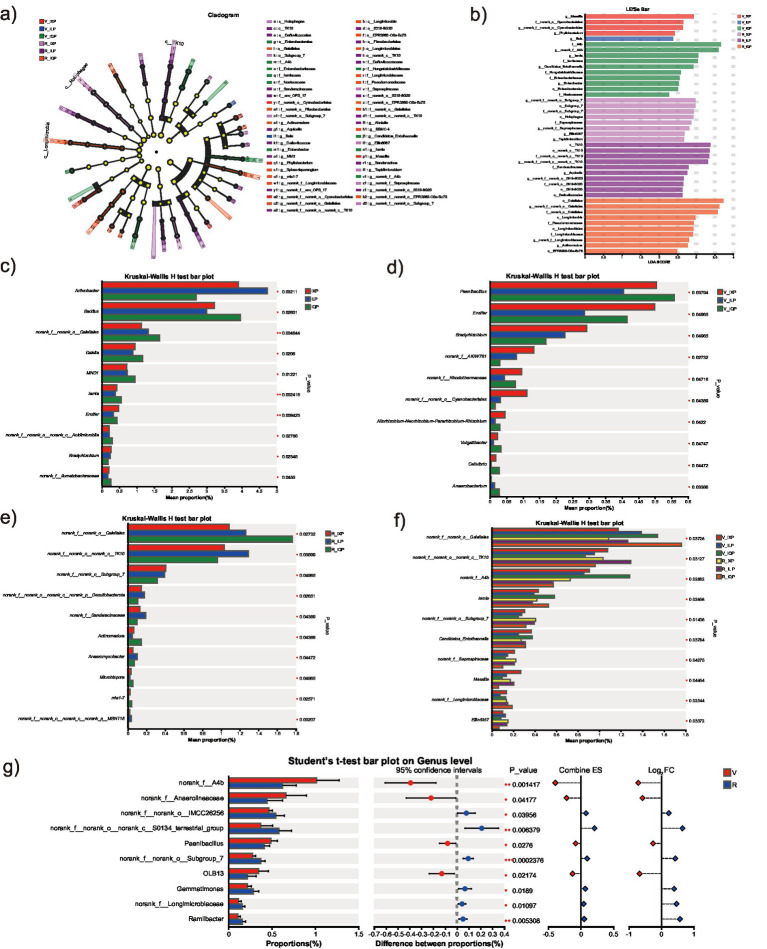
Differential bacteria among treatments. **(a)** LEfSe. **(b)** LDA. **(c)** Different genera among three quinoa cultivar conditions. **(d)** Different genera among the V stage of three quinoa cultivar conditions. **(e)** Different genera among the R stage of three quinoa cultivar conditions. **(f)** Different genera among all treatments. **(g)** Different genera between two stages.

Significantly different genera showed high abundance among different treatments, as determined by LEfSe analysis (Figures 4a,b). One family and three genera were enriched in the soil of V_IXP; one genus was enriched in the soil of V_ILP; one order, five families, and four genera were enriched in the soil of V_IQP; one order, two families, and four genera were enriched in the soil of R_IXP; one class, three orders, three families, and three genera were enriched in the soil of R_ILP; and one class, three orders, three families, and three genera were enriched in the soil of R_IQP. The most significant differences among treatments were found for the relative abundance of Proteobacteria, followed by Actinobacteriota.

The inter-group difference significance test was conducted to analyze differences in microbial composition between groups and to identify microorganisms with significant differences. The top 10 core differential genera in relative abundance are shown in Figures 4c–g. Regarding the three cultivars, the top three significantly different genera in terms of relative abundance were *Arthrobacter, Bacillus,* and *norank_f__norank_o__Gaiellales*. For the V stage of the three cultivars, the genera with the most significant differences in abundance were *Paenibacillus, Ensifer,* and *Bradyrhizobium*, whereas for the R stage, the genera *norank_f__norank_o__Gaiellales, norank_f__norank_o__norank_c__TK10,* and *norank_f__norank_o__Subgroup_7* showed the most significant differences among cultivars. For all treatments, the genera *norank_f__norank_o__Gaiellales, norank_f__norank_o__norank_c__TK10,* and *norank_f__A4b* exhibited the most significant differences, whereas *norank_f__A4b, norank_f__Anaerolineaceae,* and *norank_f__norank_o__IMCC26256* had the most significant differences in relative abundance between the two developmental stages.

### 3.5 Relationship between environmental factors and the microbial community data matrix

**Figure 5:**
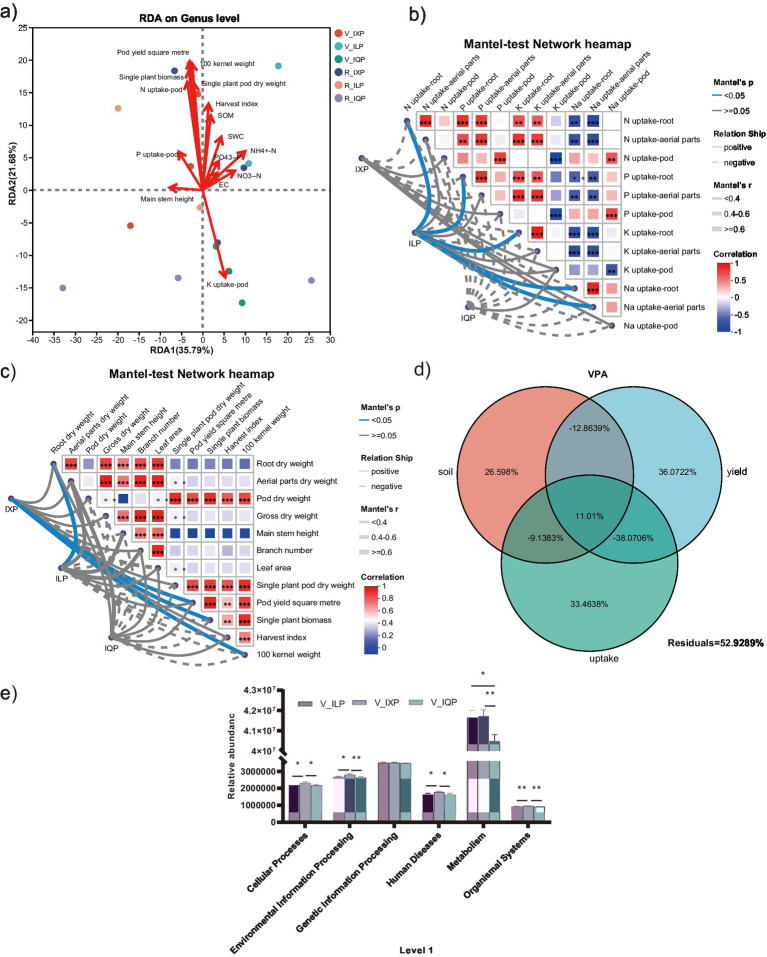
**(a)** RDA of the relationship between sample distribution and environmental factors in all treatments. **(b)** Mantel-test of uptake under three quinoa cultivar conditions. **(c)** Mantel-test of yield under three quinoa cultivar conditions. **(d)** VPA. **(e)** Level 1 of three quinoa cultivar conditions.

Redundancy analysis was conducted to reveal the relationship of the microbial community data matrix with environmental factors (Figure 5a). The first axis accounted for 35.79% of the overall variation in microbial community composition, while the second axis accounted for only 21.68% of the total variation. RDA showed that the bacterial community in the IQP was positively correlated with potassium uptake of the pod, whereas the bacterial communities in the R_ILP, V_IXP, and R_IXP treatments were positively correlated with plant growth and pod yield indicators such as pod yield/m^2^, 100-kernel weight, and single plant biomass. The bacterial community in V_ILP was positively correlated with soil indicators such as SWC, NH_4_^+^-N, and PO_4_^3−^-P.

The Mantel test network heatmap was constructed to visualize the correlation between the microbial community data matrix of the three cultivars and nutrient uptake or pod yield. The results showed that N, P, K, and Na uptake of the root and Na uptake of the aerial parts were all significantly positively correlated with the ILP (Figure 5b). In addition, pod yield/m^2^, single plant biomass, and 100-kernel weight were significantly positively correlated with the cultivar IXP condition, while root dry weight was significantly positively correlated with the ILP (Figure 5c).

Variance partitioning analysis showed that yield, uptake, and soil explained 36.07, 33.46, and 26.60% of the total variance in the rhizosphere bacterial communities, respectively, with the remaining 52.93% of the variation not explained (Figure 5d). A joint environmental effect (11.01%) explained a lower proportion of the variation in rhizosphere bacterial turnover. Among these variables, pod yield properties had a large influence on governing the bacterial turnover in the rhizosphere.

### 3.6 Association analysis

**Figure 6:**
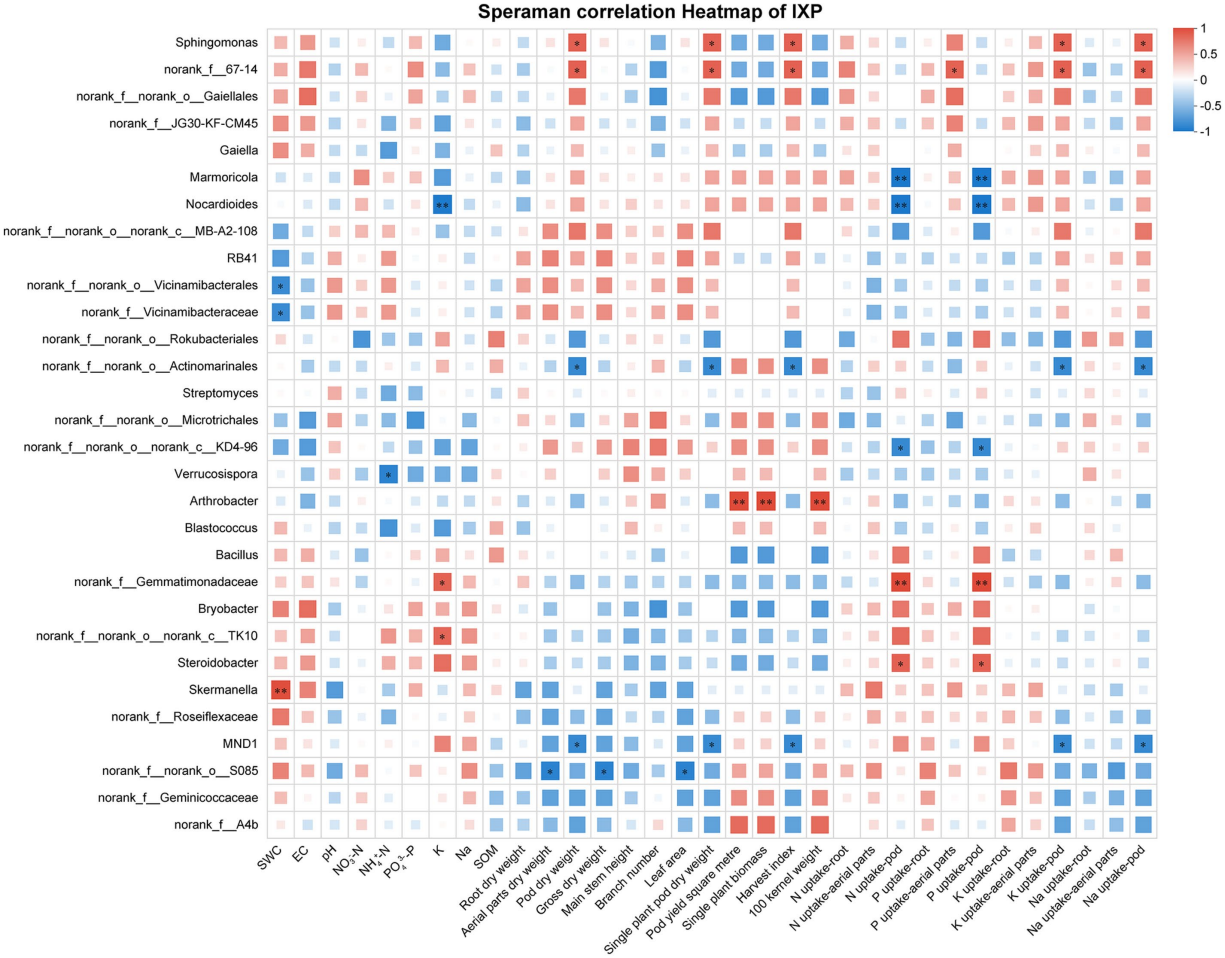
Heatmap of the correlation between IXP rhizosphere soil bacteria and environmental factors.

**Figure 7:**
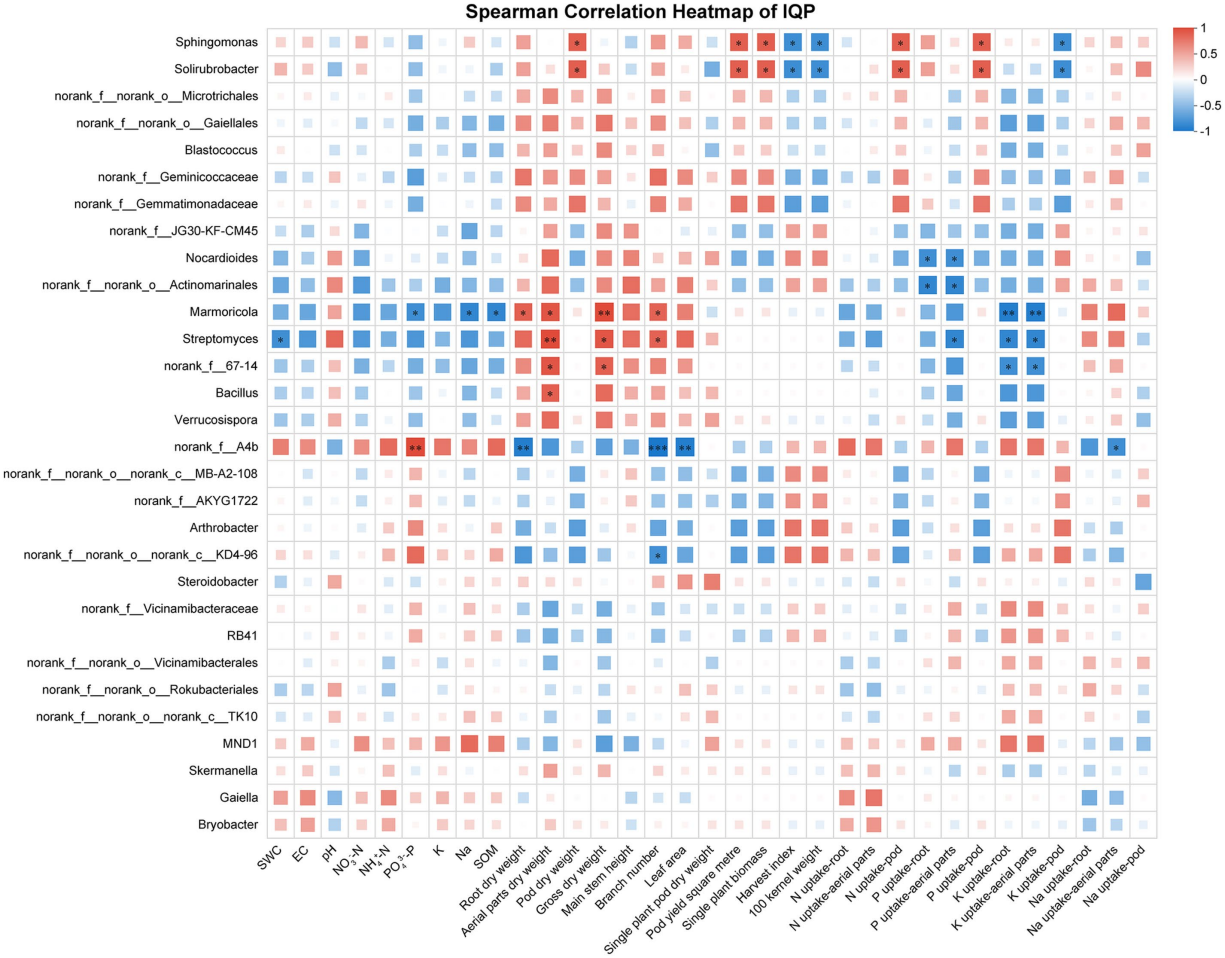
Heatmap of the correlation between IQP rhizosphere soil bacteria and environmental factors.

**Figure 8:**
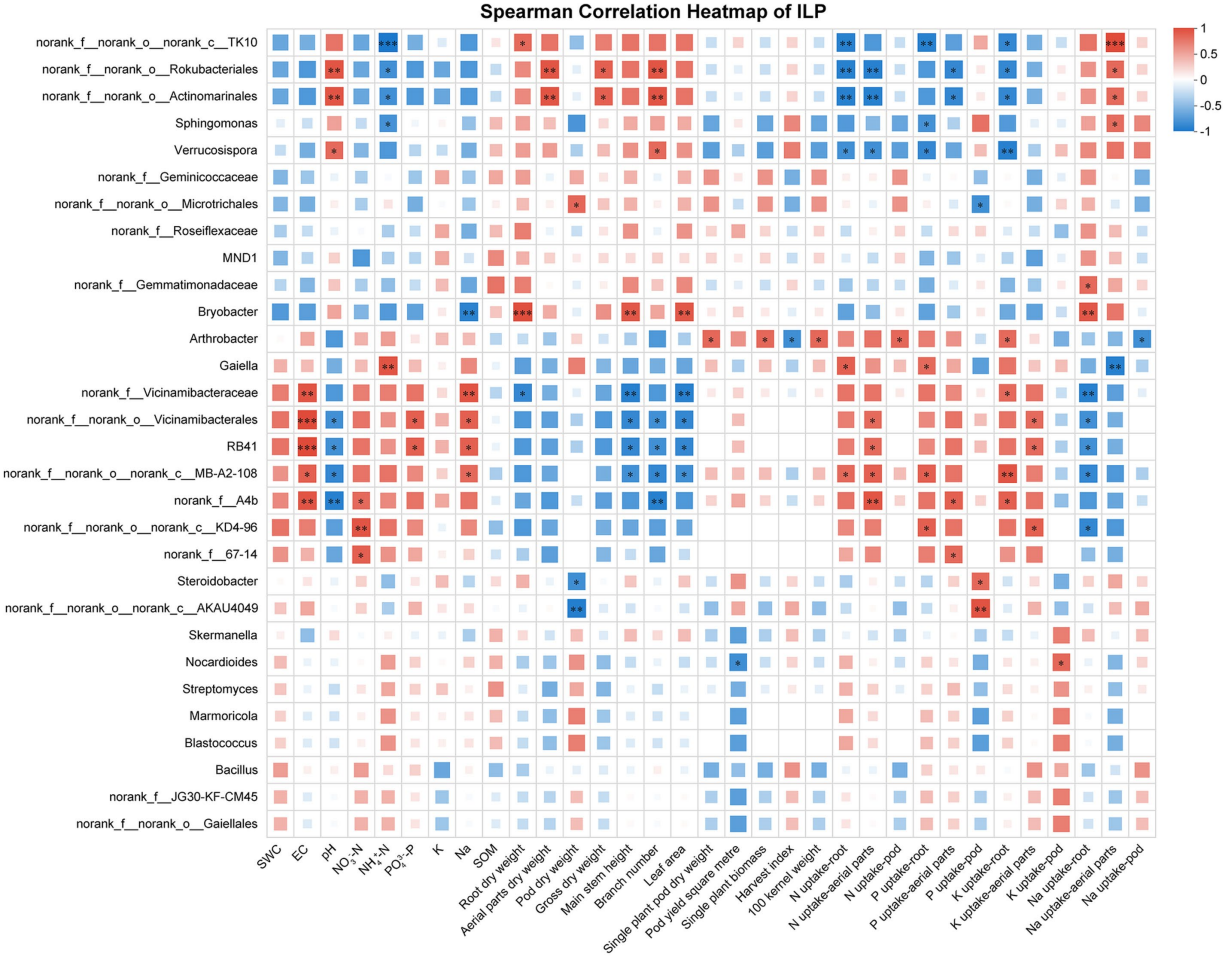
Heatmap of the correlation between ILP rhizosphere soil bacteria and environmental factors.

The correlations of different soil properties, plant vegetative, and nutrienal status with the composition of soil bacterial communities (top 30) at the genus level were visualized using a heatmap. The soil bacterial community was correlated with soil chemical properties, plant growth, pod yield, and nutrient uptake. Under the cultivar IXQ condition, the number of peanut rhizosphere bacteria was significantly correlated with peanut nutrient uptake, growth, and yield, demonstrating stronger associations than found for soil chemical indicators. The bacterial genus *norank_f__67–14 and Sphingomonas* had the most significant positive correlation, whereas *norank_f__norank_o__Actinomarinales* and *MND1* had the most significant negative correlations with plant vegetative, and nutrienal status (Figure 6). Similar to IXQ, when intercropping with the quinoa cultivar IQQ, the number of peanut rhizosphere bacteria was the most strongly significantly positively correlated with growth and yield, followed by nutrient uptake, and soil chemical indicators showed the weakest correlations. *Sphingomonas* and *Solirubrobacter* were the bacteria with the most significant positive correlation, whereas *Marmoricola, Streptomyces, and norank_f__67–14* were the genera with the most significant negative correlations with environmental indicators (Figure 7). By contrast, when intercropping with ILQ, the number of peanut rhizosphere bacteria was most strongly significantly positively correlated with nutrient uptake, followed by soil chemical indicators. The genus *norank_f__norank_o__norank_c__MB-A2-108* and *Arthrobacter* had the most significant positive correlation with environmental indicators, whereas *norank_f__norank_o__Actinomarinales, norank_f__norank_o__Rokubacteriales, and norank_f__norank_o__norank_c__TK10* had the most significant negative correlations with environmental indicators (Figure 8).

### 3.7 Potential functional consequences

The functions of microbial communities from different cultivars were predicted using PICRUSt2 on level 1 (Figure 5e). When intercropping with the quinoa cultivar IXQ, more genes were significantly associated with cellular processes, environmental information processing, human diseases, and organismal systems processes compared with the ILQ and IQQ conditions. Metabolism process was more significantly enriched among the bacteria found in the IXQ and ILQ conditions compared to intercropping with IQQ. There was no significant difference among the three cultivars with respect to genetic information processing.

## 4 Discussion

### 4.1 Dominant microorganisms and their relationship with soil, peanut growth, yield, and nutrient uptake in a quinoa-peanut intercropping system

Soil, tillage practices, and crop growth stages all exert influences on the bacterial communities associated with crop roots (Lan et al., 2025). In line with previous studies (Yang et al., 2020; Shi et al., 2021), we observed that Actinobacteria, Proteobacteria, Acidobacteriota, and Chloroflexi were the dominant bacterial phyla in all treatments. Actinobacteria play an important role in C cycling of the soil because they contain enzymes capable of degrading cellulose (Han et al., 2022), while most N-fixing microorganisms belong to Proteobacteria (Dang et al., 2020). Therefore, the richness of these bacteria in the rhizosphere may be beneficial for C and N cycles between soil and peanuts. We found that the dominant genera varied among treatments, indicating the complexity of microorganisms under the field experiment conditions. However, the bacteria with higher abundance and significant correlations with environmental factors showed some similarity among treatments. For example, members of *Arthrobacter*, a genus of Actinobacteriota that was one of the most abundant genera identified in the peanut rhizosphere, are well known as plant growth-promoting rhizobacteria (PGPR); some bacterial species in this genus have been reported to degrade PE (Han et al., 2022), inhibit plant pathogens through the antagonistic effect of secondary metabolites (Ramlawi et al., 2021; Li H. et al., 2023), improve crop yields, and enable persistence under excessive exposure to biotic and abiotic stress (Bigatton et al., 2024), such as salt stress mitigation (Fan et al., 2016; Khan et al., 2019; Safdarian et al., 2019). As one of the top three bacteria identified, *Arthrobacter* relative abundance in the rhizosphere was significantly positively correlated with pod yield/m^2^, single plant biomass, and 100-kernel weight when peanuts were intercropped with IXQ and ILQ. The genus *Sphingomonas*, another PGPR from Proteobacteria, was reported to play an important role in the decomposition of aromatic compounds, nitrogen fixation and denitrification, and the C cycle (Ci et al., 2021; Xu et al., 2023), along with a vital role in enhancement of plant tolerance to salt (Sun et al., 2022). Our findings are partially consistent with these previous reports, as *Sphingomonas* was significantly positively correlated with the dry weight, nitrogen uptake, and phosphorus uptake of the pod when peanuts were intercropped with IXQ and IQQ. Although there are relatively fewer studies involving *norank_f__67–14*, there were reports indicating that it was sensitive to temperature (Fang et al., 2021), and associated with improved pumpkin size (Zhu et al., 2024). Consistently, we found that *norank_f__67–14* was significantly positively correlated with growth and yield indicators, including gross dry weight, pod dry weight, and harvest index, in the peanut rhizosphere soil intercropped with both IXQ and IQQ. In addition, *norank_f__67–14* was significantly positively correlated with phosphorus uptake of the aerial parts of peanut in the rhizosphere soil intercropped with both IXQ and ILQ, which is in accordance with a previous report indicating that *norank_f__67–14* was positively correlated with Available Phosphorus (Liu et al., 2022). Previous studies have indicated that assembly processes in community assembly significantly influence microbial communities (Wang et al., 2023). Therefore, investigating the roles of biotic factors in driving community assembly is crucial for gaining a deeper understanding of biodiversity and functions (Jiao et al., 2022). Stochastic processes tend to be more important than deterministic processes for microbial community assembly at small scales (Zhou et al., 2022; Lv et al., 2023). Indeed, in this study, all processes were dominated by stochastic processes, especially in the microbial assembly of peanut rhizosphere soil intercropped with the quinoa cultivar IXQ and in the R stage.

### 4.2 Effects of intercropping different quinoa cultivars on Na^+^ distribution in peanuts

In most crop plants, Na^+^ is the primary cause of ion toxicity and the Na^+^ concentration is critical for salt tolerance (Chinnusamy et al., 2006). Therefore, we evaluated the accumulation and distribution of Na^+^ in peanuts under various intercropping conditions and stages. Salt stress causes high Na^+^ accumulation in peanut (Liu et al., 2023). Consistent with these findings, we observed that peanuts growing on saline-alkali soil could readily accumulate Na from the soil, and the root is the main organ for storing Na in peanut. Salinity tolerance differs across various growth stages, with greater sensitivity to salt in the early stages than in the later stages (Hussain et al., 2018). Consistently, we found that the Na concentration in both the roots and the aerial parts of the plant were higher in the R stage than in the V stage. According to Tester and Davenport (2003), the roots tend to maintain fairly constant levels of NaCl over time in saline conditions, and the plant can regulate NaCl levels by exporting salt to the soil or to the shoot. Similar results were obtained by us that there was no significant difference in the increase of Na concentration in roots among the three cultivars between the two stages. Significant differences among cultivars were attributed to the aerial parts and pods. The accumulation of Na in plants depends on the genotype and the environment (Tester and Davenport, 2003; Mohammadi et al., 2022). Shabala et al. (2013) found that more tolerant varieties accumulated more Na when not exposed to extreme salinity conditions. Quinoa is able to absorb a great amount of salt during the overlapping period in an intercropping system (Liang et al., 2025). Consistent with these findings, we observed that peanut intercropping with IXQ resulted in significantly more Na enriched in the plant, compared to that of the ILQ and IQQ conditions, above results may be due to the smaller biomass of IXQ, which absorbs less Na from the soil, resulting in a higher Na concentration in intercropping peanut IXP compared to ILP and IQP. However, despite IXP’s higher salt uptake, peanut yield remained unaffected, indicating that Na levels in this study did not reach a critical level that would detrimental to peanuts. Furthermore, the relatively shorter stature and growth cycle of quinoa plants when intercropped with IXQ allowed peanuts to receive more sunlight, resulting in higher yields.

### 4.3 Agronomic traits of quinoa variety IXQ suitable for intercropping with peanuts

The yields and productivity of intercropping are closely linked to the choice of intercropping species or cultivars (Namatsheve et al., 2020), and there has been increased research interest in specific intercropping breeding strategies in recent years (Moore et al., 2022; Dubey et al., 2024). Previous reports show substantial variations in the impacts of different cultivars on the yield of intercropping partners. For example, intercropping with the faba bean variety Gora significantly produced greater total grain yields of maize than obtained when intercropping with the faba bean cultivar Moti (Dehghanian et al., 2024; Gidey et al., 2024). However, intercropping with two different cowpea cultivars had no effect on the millet aboveground biomass (Senghor et al., 2023). In the present study, there were significant differences in the yield of peanuts intercropped with three quinoa cultivars, with the cultivar IXQ resulting in the highest yield. There are various potential reasons for this effect. Crucially, differences in light distribution and quality in the microclimatic environment of the crop canopy represent one primary factor. Light is critical for crop photosynthesis and yield, which is equally true for complex intercropping systems (Lu et al., 2023). The height of the high-position crop IXQ was the lowest among the three tested quinoa cultivars, thereby increasing in the effective photosynthetic radiation reaching the low-position crop canopy, additionally, IXQ’s shortest growth period also implies the shortest symbiotic period with peanuts, increasing the period of unobstructed sunlight for the peanuts. The combined effect ultimately resulting in the higher yield of intercropped peanut.

### 4.4 Microbial characteristics of quinoa variety IXQ suitable for intercropping with peanuts

The differences in rhizosphere microenvironment are also a very important factor affecting crop growth and yield. Plants actively regulate the rhizosphere environment by releasing root exudates into the soil, recruiting diverse microorganisms to the root zone under the guidance of these exudates (Ma et al., 2022). Plant species, growth stages, environmental factors, and microorganisms are the main factors affecting root exudates (Meng et al., 2025). Different quinoa varieties exhibit distinct rhizosphere exudates and microbial communities (Maestro-Gaitán et al., 2023). During intercropping, the close physical proximity of the roots between two crops may lead to the mixing of microbial communities between the two plant species within 28 days after sowing, and the presence of PGPR in the intercropping system would promote a favorable exchange between the two crops with an overall positive impact on growth (Chamkhi et al., 2022). Consequently, in this study, significant differences in peanut rhizosphere bacteria were also observed when intercropping different quinoa varieties, IXP significantly enriched certain bacteria. Our LEfSe and differential bacteria analyses indicated that the relative abundances of the genera *Ensifer*, *Bradyrhizobium*, and *Massilia* in the V stage and the abundance of *norank_f__norank_o__subgroup-7* in the R stage were significantly higher under IXQ intercropping than with the other two quinoa cultivars. *Bradyrhizobium* is a well-known rhizobia belonging to Proteobacteria that has a long history of use in commercial inoculants (Jovino et al., 2022). The interaction between isolates of *Bradyrhizobium* and metabolites from the peanut root helps to initiate nodulation and improve soil N availability, thereby significantly promoting peanut growth and yield of the pod (Ahsan et al., 2023; Qiao et al., 2024). *Ensifer*, another fast-growing rhizobia, has been identified in saline-alkaline soils in numerous studies (Habibi et al., 2017). In addition to playing a role in host plant growth (Wu et al., 2022; Ali et al., 2024), *Ensifer* was also reported as a potential candidate for the biocontrol of plant disease and to promote the proliferation of the AM fungus (Yang, 2019; Wiriya et al., 2020). *Massilia*, a copiotrophic bacterium belonging to Proteobacteria (Su et al., 2020), can synthesize various secondary metabolites and enzymes, promote organic matter decomposition and nitrogen cycling, facilitate phosphorus solubilization and degradation, enhance heavy metal tolerance, increase crop yield, and play an important role in soil remediation and amelioration (Zheng et al., 2024; Ding J. et al., 2025). The genus *norank_f__norank_o__ subgroup-7* of Acidobacteriota has been reported as a beneficial functional bacterium related to crop yield, nitrogen uptake (Fang et al., 2025), nutrient availability (Navarrete et al., 2015), ecological function of the soil, and soil microecology protection (Chen et al., 2023c). The above functional flora likely contribute to the high yield of peanut intercropped with quinoa IXQ. Additionally, regarding microbial functional characteristics, the significantly higher activity of IXP across five Level 1 processes likely contributes to its high productivity. Particularly in metabolic activity, IXP exhibited the highest levels while IQP showed the lowest, mirroring the yield ranking. This demonstrates a close correlation between microbial functions and the growth and yield of intercropped peanuts. Furthermore, during bacterial assembly, species from the IXP group exhibited the highest degree of stochastic processes at both two stages. Whether this characteristic is closely linked to the high productivity of IXP requires further research to confirm. Finally, pod yield properties were more related to the bacterial turnover in the rhizosphere than plant nutrient uptake and soil factors, understanding the specific reasons contributing to this difference necessitates further research.

## 5 Conclusion

Significant differences in the correlations between peanut rhizosphere microorganisms and soil chemical properties, peanut growth, nutrient uptake, and pod yield were found when intercropping with three different quinoa cultivars. Actinobacteriota and Proteobacteria were the most dominant bacterial phyla, while *norank_f__Geminicoccaceae*, *norank_f__norank_o__Vicinamibacterales*, and *Arthrobacter* were the dominant bacterial genera identified among all treatments. The correlations between peanut rhizosphere microorganisms and yield were higher when intercropping with IXQ than with other quinoa cultivars, whereas intercropping with ILQ resulted in a higher correlation of microorganisms with soil and nutrient absorption. The relative abundance of microorganisms involved in metabolism processes was significantly higher in both IXP and ILP than IQP. Intercropping the quinoa cultivar IXQ had a significantly greater effect in facilitating peanuts to accumulate Na from the soil compared to the other two cultivars, ILQ and IQQ, without affecting the yield. Therefore, IXQ appears to be a more suitable cultivar choice for intercropping with peanuts in saline-alkali soil. In a quinoa-peanut intercropping system, selecting quinoa cultivars with shorter plant height and shorter growth cycle is recommended to enhance peanut yield. Overall, this study can provide a valuable reference for the rational and sustainable utilization of saline-alkali land.

## Data Availability

The datasets presented in this study can be found in online repositories. The names of the repository/repositories and accession number(s) can be found in the article/Supplementary material.

## Footnotes

1
www.majorbio.com

2
http://galaxy.biobakery.org/
