# Supplementary material for: Effects of intercropping different quinoa cultivars on peanut rhizosphere microorganisms and yield in saline-alkali soil
Source: Front Microbiol. 2025 Dec 17;16:1729353. doi: 10.3389/fmicb.2025.1729353 (PMC12753954; doi:10.3389/fmicb.2025.1729353)
Supplement: Supplementary file 1 [file Data_Sheet_1.docx]

**Table S1 Intercropping details**

|  | Experiment details |
| --- | --- |
| Intercropping | Each plot was size 140 m^2^ (14 m×10 m), with 8 rows of peanut. Seeds of the commercial peanut cultivar Huayu 25 were generously provided by Shandong Peanut Research Institute, Shandong Academy of Agricultural Sciences (Qingdao, China).In the relay intercropping systems, three variety of quinoa were all sowed first in March 15 with a row space of 50 cm and plant distance of 25 cm, and peanut was sowed in May 8 with two rows on each ridge, with a ridge width of 50 cm and furrow width of 30 cm. Row space on the same ridge was 30 cm and plant distance was 15 cm. Each intercropping zone consisted of four rows of peanut and three rows of quinoa. |
| Field management | Peanuts in different intercropping systems were harvested on tember 12. The quinoa variety of Xingli-3 was harvested on June 26, and the quinoa varieties of Longli-4 and Qingli-2 were harvested on July 15. In each experimental plot, rotten chicken manure (225 kg·ha-1) was applied in the winter of the year before sowing. In addition, compound fertilizer (N-P_2_O_5_-K_2_O: 15-15-15) of 600 kg·hm^-2^ was applied in quinoa planting belt as a base fertilizer before the sowing of quinoa, and nitrogen (N) 90 kg·hm^-2^, phosphorus (P_2_O_5_) 120 kg·hm^-2^, potassium (K_2_O) 150 kg·hm^-2^ and slow-release nitrogen fertilizer 90 kg·hm^-2^ were applied in peanut planting belt as a base fertilizer before the sowing of peanut. The total amount of irrigation water in each cropping pattern was the same during the growing season of peanut with irrigation in each treatment. All the other cultivation and management measures were the same during the growing stage of peanut plants. |
| Plant sample | Four uniform plants were sampled at the vegetative (V) stage and reproductive (R) stage of peanuts in each treatment to determine the main stem height, branch number and leaf area per plant. |
| Plant dry weight and nutrient accumulation | Plant samples were categorized as roots, stem and leaves, or pods. Samples were dried at 105℃ for 30 min to kill living plant tissue. Next, they were dried at 75℃ to a constant weight and then the dry weights of each part were immediately recorded. The dry biomass of the entire plant was recorded. The samples were finely ground to powder and digested with H2SO4-H2O2 as separate plant parts. Nitrogen (N) concetration was assayed using the micro Kjeldahl analysis (Barbano,1991). Phosphorus (P) concetration was determined by a flow analyzer according to the manufacturer’s instructions (Khashi, 2021). Potassium (K) concetration was measured using a flame photometer as described by K Chakraborty, D Bhaduri, HN Meena and K Kalariya (Chakraborty, 2016). N , P or K accumulation in the different [plant organs](https://www.sciencedirect.com/topics/agricultural-and-biological-sciences/plant-organs) was determined from the product of the N, P or K concetration and the biomass weight. The determination of leaf area is carried out by punching holes in the leaves of peanut. One leaf disc with a diameter of 1.0 cm was punched for each of the third leaf from the top of peanut plants, leaf discs and all leaf samples were dried at 75 °C to constant weight. Then, the dry weight of leaf discs and all leaf samples was weighed and recorded. Leaf area (LA) of plant was calculated as follows: LA=LAdisc/DMdisc×DMtotal (cm^2^). |
| Determination of yield and yield component | The pods from peanut plants of 8 square meters in the two borders and two inner rows in different intercropping systems, were manually harvested, simultaneously the pod number per plant was recorded. After sun-dried for 15 days, the pod weight per plant, biomass per plant and pod yield were weighed and recorded. Harvest index is equal to the ratio of pod weight to biomass of peanut. At the same time, 100-kernel-weight was determined. |


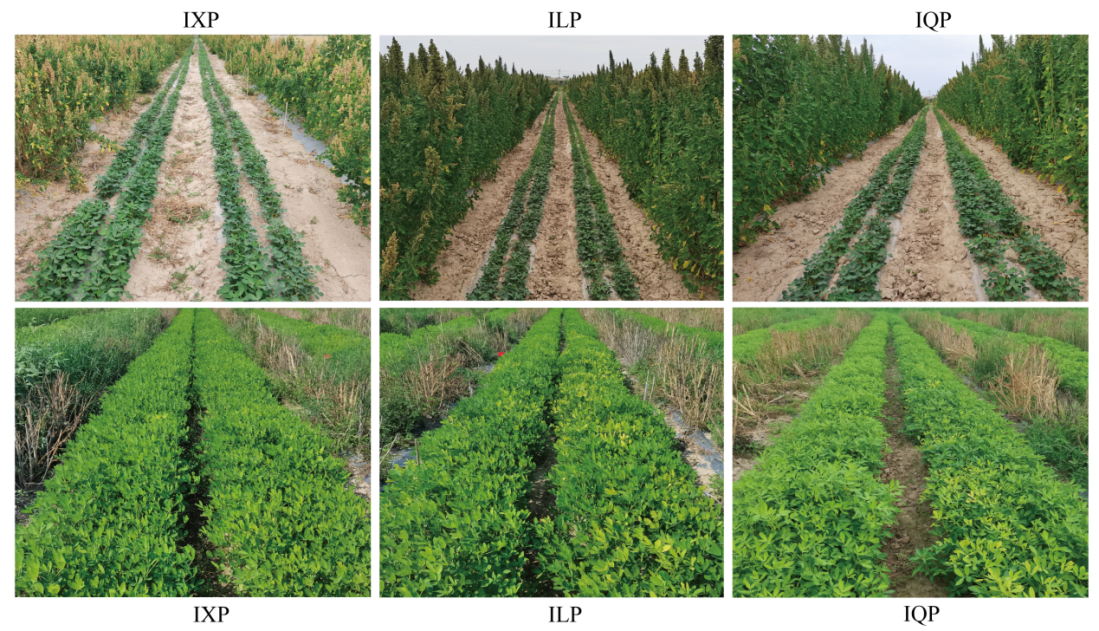


Fig. S1 Pictures of three different quinoa varieties intercropped with peanuts at different growth periods

Table S2 The plant growth characteristics of different quinoa varieties in the experiment

| Treatment | Plant height  (cm) | Growth cycle  (d) | Sowing time | Harvest time | Co-growth days (d) |
| --- | --- | --- | --- | --- | --- |
| IXQ | 125 | 100 | 3/10 | 6/20 | 45 |
| ILQ | 165 | 110 | 3/10 | 6/30 | 55 |
| IQQ | 185 | 120 | 3/10 | 7/10 | 65 |

**Table S3 PCR details**

|  | experiment details |
| --- | --- |
| PCR mixtures | The PCR mixtures contained 4 μL of 5x TransStartFastPfu buffer, 2 μL of 2.5 mM dNTPs, 0.8 μL of each primer (5 μM each), 0.4 μL of *TransStart*FastPfu DNA Polymerase, 10 ng template DNA, adding ddH_2_O to a final volume of 20 μL. All reactions were performed in triplicate. |
| PCR cycling conditions | PCR cycling conditions included an initial denaturation at 95°C for 3 min, 27 cycles of denaturing at 95°C for 30 s, annealing at 55°C for 30 s, and extension at 72°C for 45 s, followed by a single extension at 72°C for 10 min and a continued hold at 4°C. |
| Splicing and quality control | Raw sequence reads were demultiplexed, quality-filtered by fastp version 0.20.0, and merged by FLASH version 1.2.7. Operational taxonomic units (OTUs), with a 97% similarity cut-off, were clustered using UPARSE v.7.1, and chimeric sequences were identified and removed. The taxonomy of each OTU representative sequence was analyzed by RDP Classifier v.2.2 against the 16S rRNA database using a confidence threshold of 0.7. |

Table S4 Diversity index

|  | sobs | shannon | simpson | ace | chao | coverage |
| --- | --- | --- | --- | --- | --- | --- |
| R_ILP | 2108.3±64.9a | 6.42±0.12a | 0.0056±0.00135a | 2866.58±72.66ab | 2864.36±30.78ab | 0.9626±0.0008ab |
| R_IQP | 2025.7±24.1a | 6.38±0.03a | 0.0046±0.00035a | 2837.57±47.78ab | 2822.04±52.47ab | 0.9626±0.0007ab |
| R_IXP | 2129.7±64a | 6.46±0.12a | 0.005±0.00158a | 2984.9±57.13a | 2988.99±71.42a | 0.9606±0.0013b |
| V_ILP | 2022.3±78.4a | 6.31±0.04a | 0.0064±0.00091a | 2844.41±129.11ab | 2824.56±170.95ab | 0.9624±0.0021ab |
| V_IQP | 2022.7±101.9a | 6.43±0.09a | 0.0043±0.00076a | 2771.13±135.99b | 2718.13±156.35b | 0.9643±0.0022a |
| V_IXP | 2134±80.3a | 6.45±0.03a | 0.005±0.00083a | 3020±128.87a | 3018.18±81.62a | 0.9601±0.0019b |


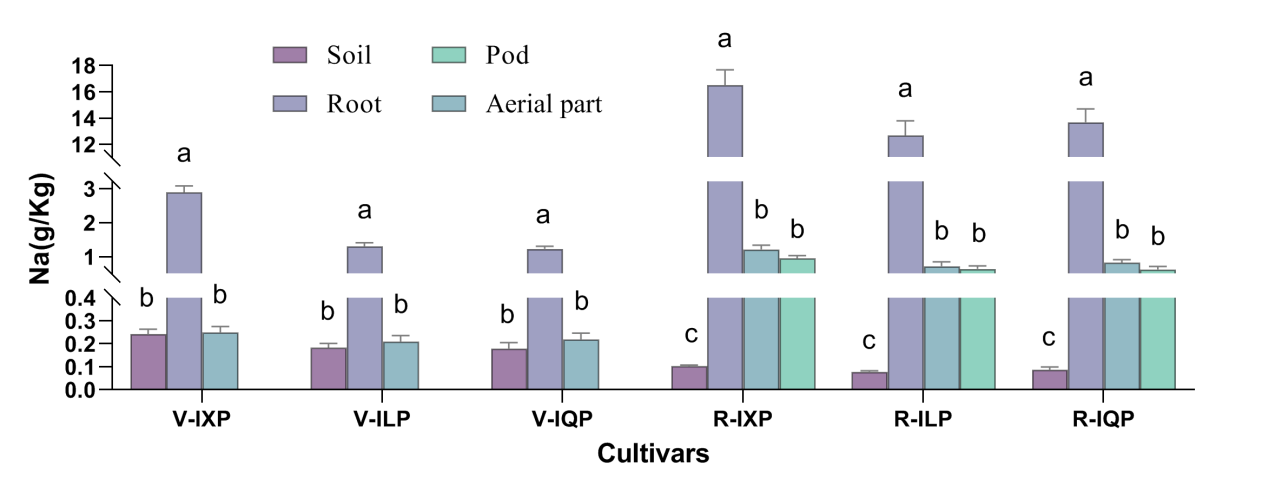
Figure S2 Na concetration of soil and different parts


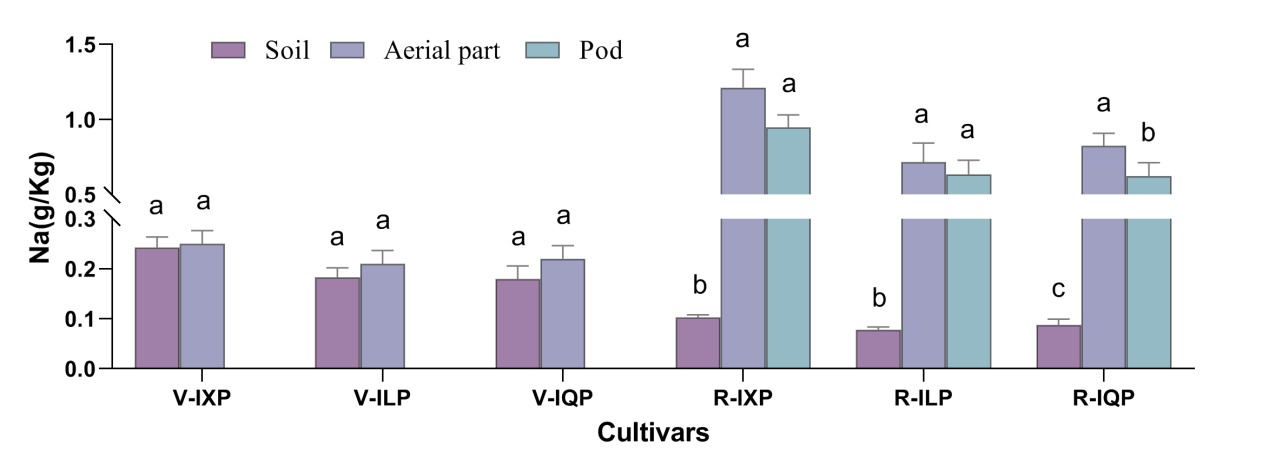


Figure S 3 Na concetration of soil and different parts without root


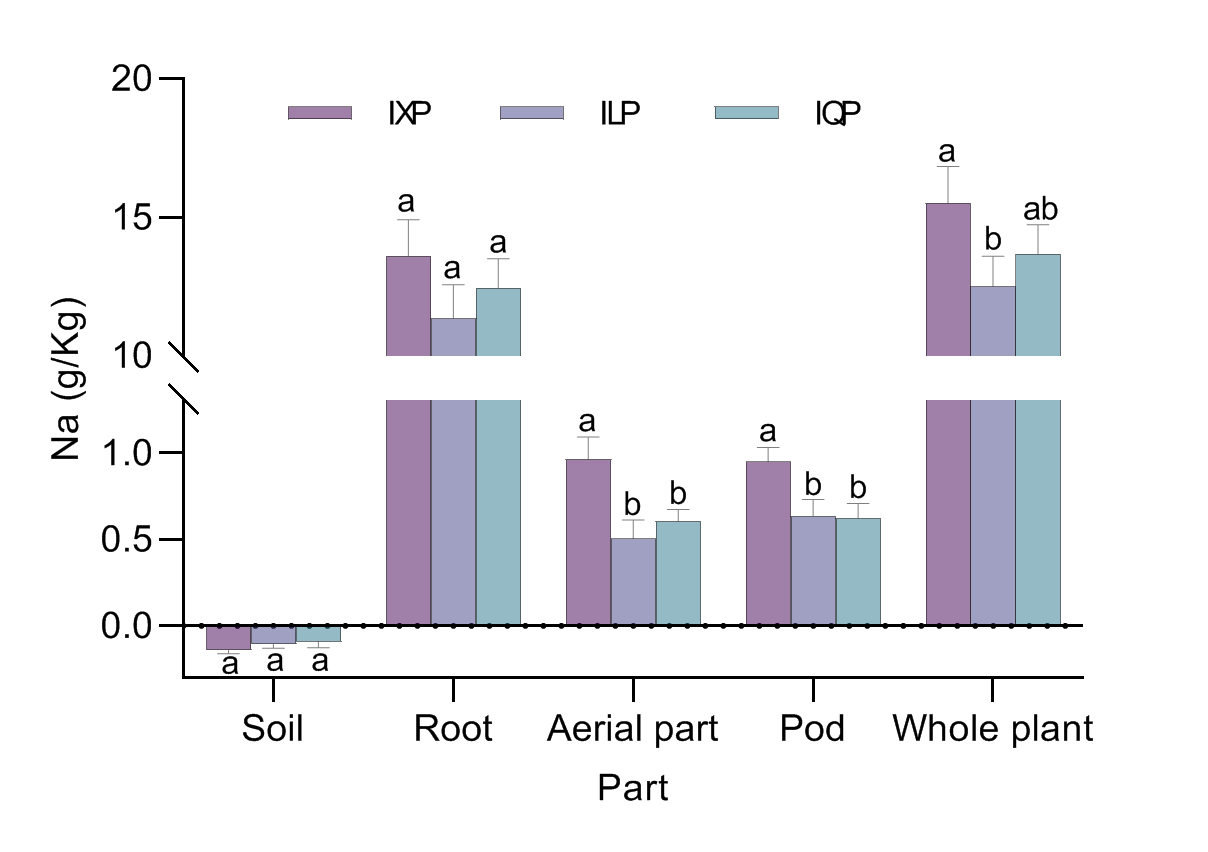


Figure S4 Na concetration change of soil and different peanut parts between two stage


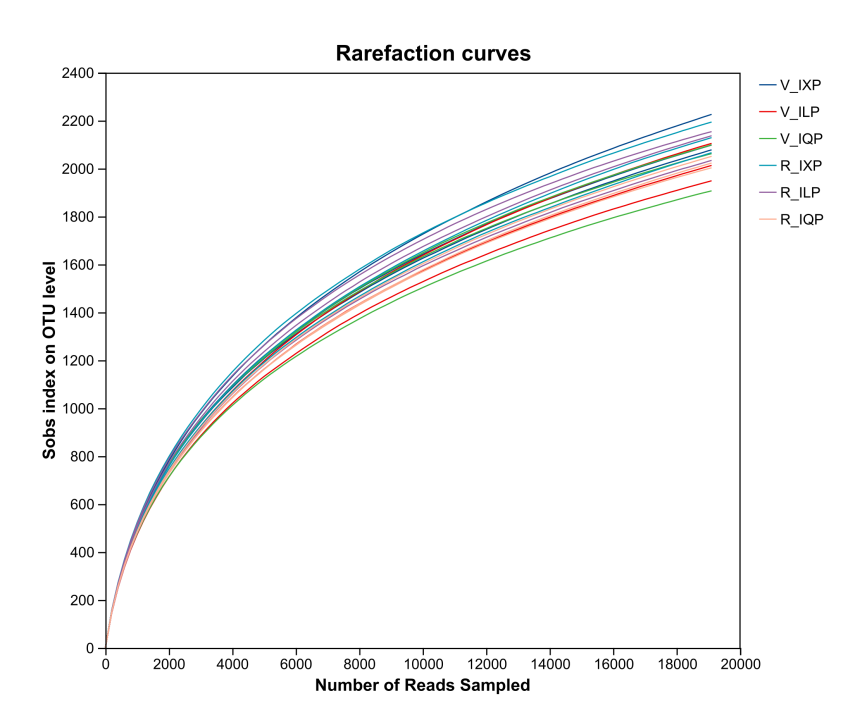


Figure S5 Rarefaction curves


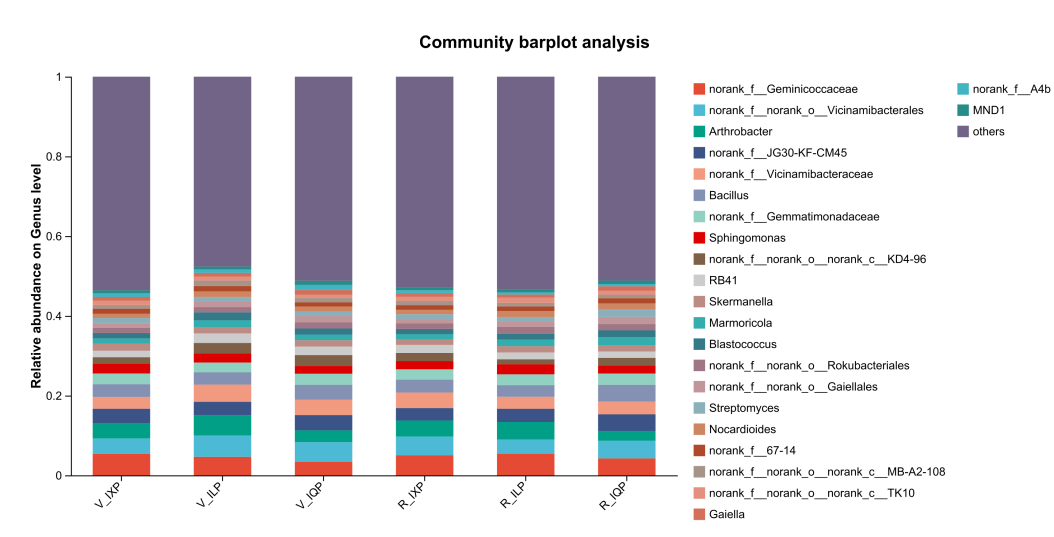


Figure S6 Bar on genus level


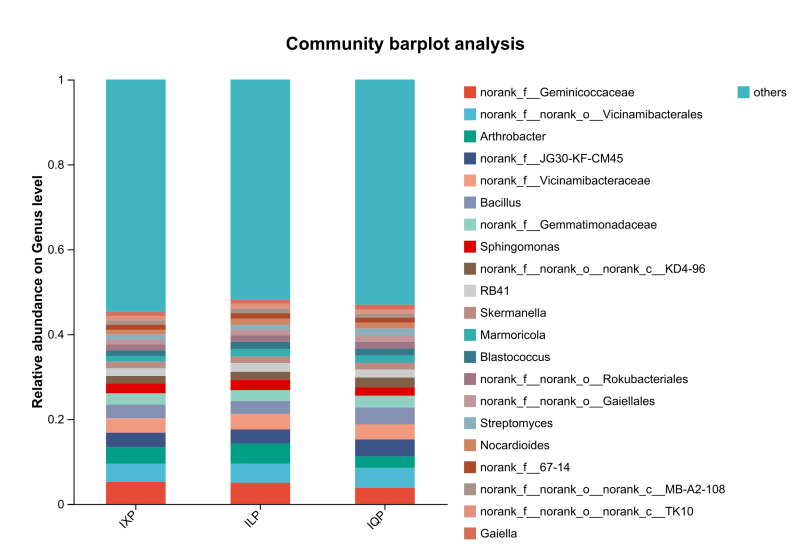


Figure S7 Bar on genus level


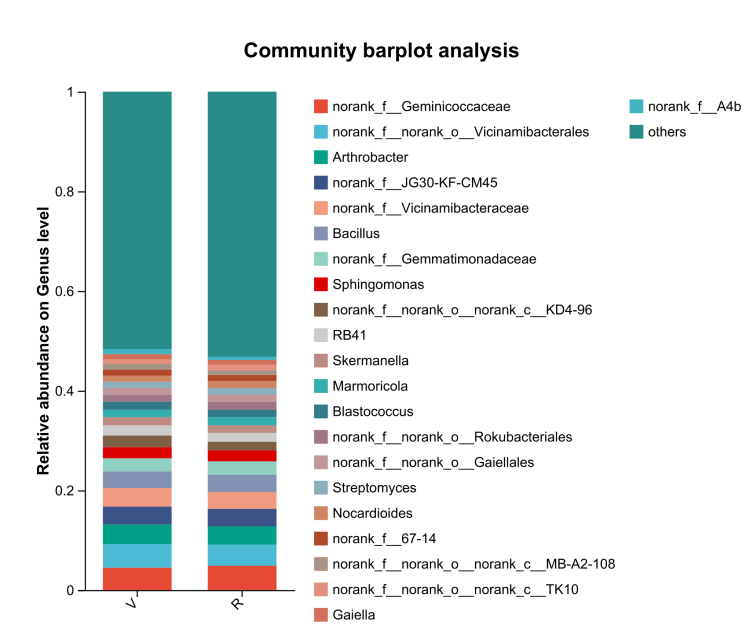


Figure S8 Bar on genus level
